# Anti-Inflammatory Activity of *Mandragora autumnalis* Ethanolic Extract: In Vitro and Cellular Mechanistic Insights

**DOI:** 10.3390/ph19030483

**Published:** 2026-03-15

**Authors:** Ghosoon Albahri, Heba Hellany, Adnan Badran, Rami Abdel-Rahem, Mayyas Al-Remawi, Mohamad Alame, Akram Hijazi, Mikhael Bechelany, Elias Baydoun

**Affiliations:** 1 Faculty of Arts and Sciences, American University of Beirut, Beirut 1107, Lebanon or ghosoon.albahri.1@ul.edu.lb (G.A.); he115@aub.edu.lb (H.H.); 2 Doctoral School of Science and Technology-Platform of Research and Analysis in Environmental Sciences (EDST-PRASE), Beirut 1107, Lebanon; alamefs@hotmail.com (M.A.); akram.hijazi@ul.edu.lb (A.H.); 3 Department of Biology, University of Jordan, Amman 11942, Jordan; adnan.badran@ju.edu.jo; 4 Department of Chemistry, University of Petra, Amman 11196, Jordan; rabdelrahem@uop.edu.jo; 5 Faculty of Pharmacy & Medical Sciences, University of Petra, Amman 19328, Jordan; malremawi@uop.edu.jo; 6 Institut Européen des Membranes, IEM, UMR-5635, University Montpellier, ENSCM, CNRS, Place Eugene Bataillon, 34095 Montpellier, France

**Keywords:** *Mandragora autumnalis*, inflammation, nitric oxide, cytokine modulation, membrane stabilization, MAPK signaling pathways

## Abstract

Plant-based remedies have been employed since ancient times to manage and prevent a wide range of diseases. **Background:** Their bioactive constituents provide natural alternatives to synthetic anti-inflammatory drugs, often with reduced toxicity and multiple biological benefits. *Mandragora autumnalis*, a medicinal plant rich in secondary metabolites, has not been extensively investigated for its anti-inflammatory properties. **Methods**: The anti-inflammatory activity of *Mandragora autumnalis* ethanolic extract (MAE) was evaluated using in vitro biochemical assays, including heat-induced protein denaturation (casein and bovine serum albumin) and red blood cell membrane stabilization. Cellular anti-inflammatory effects were assessed in LPS-stimulated RAW 264.7 macrophages by measuring nitric oxide production, pro-inflammatory cytokine levels, macrophage migration, gene and protein expression of inflammatory mediators, and the activation status of NF-κB, STAT3, and MAPK signaling pathways. **Results:** Preliminary screening revealed that MAE effectively inhibited heat-induced protein denaturation (casein and bovine serum albumin) and heat-induced red blood cell (RBC) hemolysis, indicating strong protein- and membrane-stabilizing properties associated with anti-inflammatory activity. In lipopolysaccharide (LPS)-stimulated RAW 264.7 macrophages, MAE markedly suppressed the inflammatory response by downregulating the expression of inducible nitric oxide synthase (iNOS) and cyclooxygenase-2 (COX-2), leading to a significant reduction in nitric oxide (NO) production and pro-inflammatory cytokines, including tumor necrosis factor-α (TNF-α) and interleukin-6 (IL-6). Furthermore, MAE inhibited macrophage migration and attenuated key inflammatory signaling pathways, notably nuclear factor kappa B (NF-κB), signal transducer and activator of transcription 3 (STAT3), and mitogen-activated protein kinases (MAPKs). Molecular docking analysis was conducted to explore the potential interactions between the most abundant chemical compounds and key proteins involved in inflammatory signaling pathways, including ERK and iNOS. **Conclusions:** Overall, these results indicate that MAE exhibits strong anti-inflammatory activity by stabilizing proteins, protecting cellular membranes, and inhibiting key inflammatory mediators and signaling pathways. These findings highlight its potential as a natural therapeutic candidate for the prevention and management of chronic inflammation-related disorders, such as arthritis, cardiovascular diseases, and cancer. However, further mechanistic investigations and in vivo studies are required to confirm its therapeutic potential and clinical relevance.

## 1. Introduction

The immune system’s natural physiological response to infection, tissue damage, or exposure to toxic stimuli is inflammation [1]. As a defense mechanism, it aids in the removal of infections or damaged cells, initiating the healing process [2]. Acute inflammation is a rapid, short-term response that usually subsides once the underlying cause is removed. It is characterized by redness, heat, swelling, pain, and a temporary loss of function in the affected tissue [3]. On the other hand, chronic inflammation is a protracted reaction that can last anywhere from months to years [4,5]. It often leads to progressive tissue damage and is associated with the development of a number of chronic conditions, including multiple cancers, cardiovascular diseases, and arthritis [6]. Signaling pathways and inflammatory mediators interact in a complex way to initiate inflammation [7]. Innate immune cells, including macrophages, identify pathogen-associated molecular patterns (PAMPs) and damage-associated molecular patterns (DAMPs) in infected or injured tissues [8]. This recognition triggers intracellular signaling pathways and the generation of pro-inflammatory mediators [9]. Folk medicine has long utilized plant extracts to manage inflammation and related conditions [10]. These extracts are derived from various plant parts and contain bioactive substances that may offer therapeutic benefits. Plants are abundant in phytochemicals that influence inflammatory pathways and prevent the synthesis of pro-inflammatory enzymes and cytokines, including curcumin, gingerols, polyphenols, salicin, and boswellic acids [11]. Plant extracts have anti-inflammatory properties through a variety of mechanisms [12]. They could have an impact on the synthesis or function of pro-inflammatory mediators like cytokines, leukotrienes, and prostaglandins [7]. Significant antioxidant activity has also been demonstrated by numerous plant extracts, which reduce oxidative stress, promote the synthesis of antioxidant enzymes, and lessen inflammatory damage [13]. Additionally, these extracts can modify key signalling pathways linked to inflammation, including NF-κB, JAK-STAT, and MAPK pathways [14]. Since ancient times, the *Mandragora autumnalis* plant has been revered as one of the most important medicinal plants, serving as a herb of significant cultural value. Its historical application has been impressive. Among the many uses of *Mandragora autumnalis* are medicinal, hallucinogenic, and ovulation-promoting. The narcotic effect of *Mandragora autumnalis* is also likely due to the presence of an alternative form of alkaloids [15]. It has been shown that *Mandragora autumnalis* has narcotic effects in addition to antibacterial, antioxidant, and antitumor properties. *Mandragora* species have been found to contain several phytochemicals, including lipid-like compounds (β-sitosterol), coumarins (umbelliferone and scopoletin), alkaloids (atropine and scopolamine), and withanolides (salpichrolide C) [16]. Moreover, little is known about MAE’s anti-inflammatory properties and underlying mechanisms of action. In this work, the anti-inflammatory properties of MAE were examined in vitro against RAW 264.7 macrophages stimulated by lipopolysaccharide (LPS), with an emphasis on how it affected important inflammatory mediators and signaling pathways. In addition to reducing the production of inflammatory mediators like nitric oxide (NO), as well as the TNF-α and IL-6 cytokines, the extract was assessed for its capacity to inhibit the activation of important inducible proinflammatory enzymes like cyclooxygenase-2 (COX-2) and inducible nitric oxide synthases (iNOS). MAE’s impact on the NF-κB, STAT-3, and MAPK signaling pathways, all of which are known to be crucial for the inflammatory response and its resolution, was examined to determine the underlying mechanism by which it operates. Moreover, one of the biochemical events accompanying inflammatory conditions is protein denaturation, which can lead to loss of protein function and the generation of auto-antigens that further amplify inflammatory responses [17]. Stabilization of proteins against denaturation is therefore considered a relevant mechanism underlying the anti-inflammatory action of several pharmacological agents, including nonsteroidal anti-inflammatory drugs (NSAIDs) [17]. In vitro protein denaturation assays using model proteins such as casein and bovine serum albumin (BSA) are widely employed as preliminary screening tools to assess the anti-inflammatory potential of natural products and synthetic compounds [18]. Stabilization of cellular membranes is a key mechanism in preventing inflammatory tissue damage. Additionally, the red blood cell (RBC) membrane closely resembles the lysosomal membrane, whose destabilization during inflammation leads to the release of hydrolytic enzymes and amplification of inflammatory responses [19].

The choice of this species was primarily guided by its strong ethnomedicinal background in the Mediterranean region, where it has traditionally been used for analgesic, antispasmodic, sedative, and anti-inflammatory purposes [20]. Although the plant is known to contain tropane alkaloids such as atropine and scopolamine, which can produce toxicity and narcotic effects at high doses, these same compounds are clinically employed as anticholinergic agents under controlled medical use, illustrating the well-established principle that pharmacological activity and toxicity are dose-dependent [21]. Furthermore, beyond tropane alkaloids, *M. autumnalis* contains other secondary metabolites that may contribute to its biological activities and warrant investigation [15]. Given the growing need for novel bioactive compounds, particularly from underexplored medicinal plants within the Solanaceae family [22]. Our study evaluated the anti-inflammatory potential of *Mandragora autumnalis* ethanolic extract (MAE) in LPS-stimulated RAW 264.7 macrophages. Also, the anti-inflammatory potential of MAE was evaluated by assessing its inhibitory effect on heat-induced denaturation of casein and BSA and across a range of concentrations using the heat-induced red blood cell hemolysis assay. Molecular docking was also carried out to evaluate the potential binding interactions of most abundant chemical compounds with key proteins associated with inflammatory signaling pathways, particularly ERK and iNOS.

## 2. Results

### 2.1. MAE Maintains RAW 264.7 Macrophage Viability

**Figure 1:**
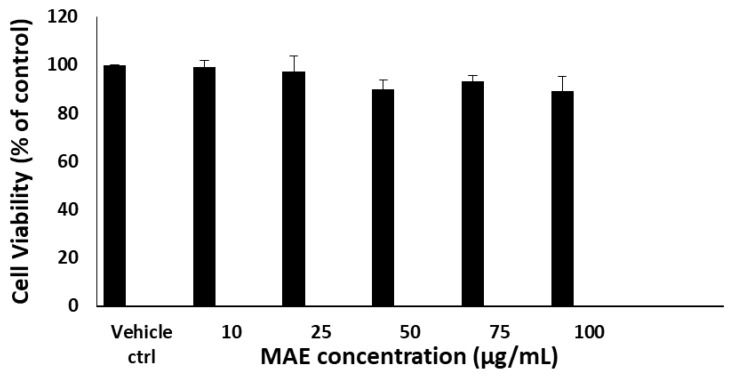
Cell viability was assessed using the metabolic-dye-based MTT assay after cells were treated for 24 h with either the vehicle control or the specified concentrations of MAE. The data are presented as a percentage of the corresponding control cells and show the mean ± SEM of three separate experiments (n = 3). Values of triplicates were presented in Table S1 in the Supplementary Materials.

The MTT colorimetric test was used to assess the effect of MAE on the viability of RAW264.7 macrophages. The cells were exposed to different concentrations of MAE (0, 10, 25, 50, 75, and 100 μg/mL) for a duration of 24 h. All concentrations of MAE did not significantly affect RAW264 viability. The lack of direct proportionality between MAE concentration and cell viability indicates that the extract does not induce a classical dose-dependent cytotoxic response within the tested range (10–100 µg/mL). As shown in Figure 1, cell viability remains consistently high, suggesting that the tested concentrations fall within a non-toxic window. Moreover, MAE concentrations of 10 and 25 µg/mL are the optimal concentrations that offer anti-inflammatory benefits while posing the least toxicity compared to other concentrations used. The anti-inflammatory potential of MAE was thus ascertained using these concentrations in later experiments.

### 2.2. MAE Inhibits LPS-Activated RAW 264.7 Macrophages’ Production of NO and iNOS Levels

**Figure 2:**
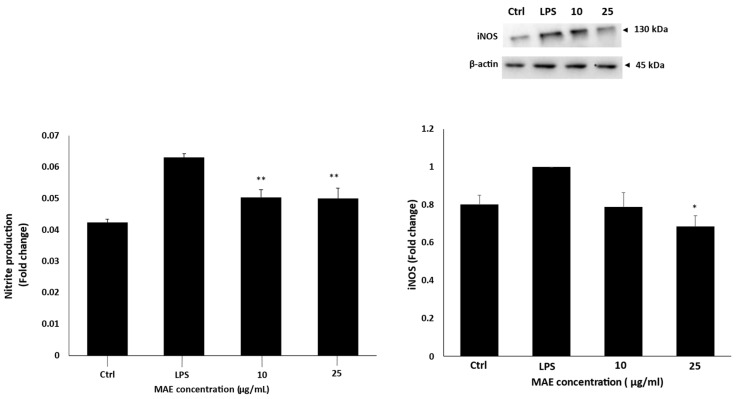
MAE prevents RAW 264.7 cells stimulated with LPS from producing NO. After 24 h, RAW 264.7 cells were either left untreated (control), treated with LPS, or treated with a combination of MAE and LPS at the specified concentrations (10 and 25 μg/mL, respectively). Western analysis was used to assess iNOS protein levels, and nitrite production was measured using a nitrite assay kit with Griess Reagent. The values are displayed as the mean ± SEM of three separate experiments and are expressed as the fold change relative to the control (** *p* < 0.01 and * *p* < 0.05). Values of triplicates were presented in Table S2 in the Supplementary Materials.

iNOS protein levels were measured in RAW 264.7 macrophages stimulated by LPS both with and without MAE treatment. MAE treatment of LPS-induced RAW 264.7 cells reduced iNOS protein levels in a concentration-dependent manner according to Western blot analysis. In comparison to the LPS-treated control, the results indicated that LPS enhanced the expression of iNOS, while 10 or 25 μg/mL MAE decreased the protein levels of iNOS by 0.78 ± 0.07-fold and 0.68 ± 0.05-fold, respectively. Additionally, MAE-treated LPS-induced RAW 264.7 cells had lower levels of nitrite, which is an indirect indicator of NO production, than untreated cells. Indeed, in comparison to the LPS control, 10 and 25 µg/mL of MAE reduced the amount of nitrite generated to 0.05 ± 0.003 times (Figure 2).

### 2.3. MAE Inhibits the Expression of Inflammatory Genes in RAW 264.7 Cells Induced by LPS

**Figure 3:**
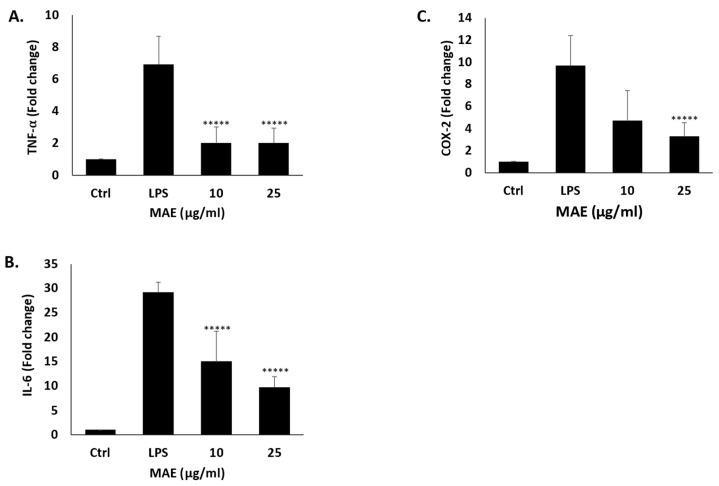
MAE prevents RAW 264.7 cells stimulated by LPS from producing TNF-α (**A**), COX-2 (**B**), and IL-6 (**C**). Over the course of 24 h, RAW 264.7 cells were either left untreated (control), treated with LPS, or treated with a combination of LPS and MAE at the specified concentrations. RT-PCR was used to assess the mRNA expression levels of the three pro-inflammatory mediators. Values are shown as the mean ± SEM of three separate experiments and are expressed as the fold change relative to the control (***** *p* < 0.00001). Values of triplicates were presented in Table S3 in the Supplementary Materials.

Using RT-PCR, we investigated how MAE affected the transcription of pro-inflammatory mediators, including COX-2, TNF-α, and IL-6. The findings demonstrated that in LPS-induced RAW 264.7 cells, treatment with MAE concentrations (10 and 25 µg/mL) significantly suppressed the transcription of the three inflammatory mediators (Figure 3). Indeed, after administering 25 µg/mL of MAE to the cells, the levels of TNF-α, COX-2, and IL-6 mRNA decreased significantly to 2 ± 0.9, 3.28 ± 1.2, and 9.7 ± 2.2 times, respectively, compared to LPS-treated control. These findings imply that MAE may have an anti-inflammatory effect by transcriptionally suppressing the production of pro-inflammatory cytokines.

### 2.4. MAE Suppresses RAW 264.7 Cell Migration in LPS-Induced Inflammation

**Figure 4:**
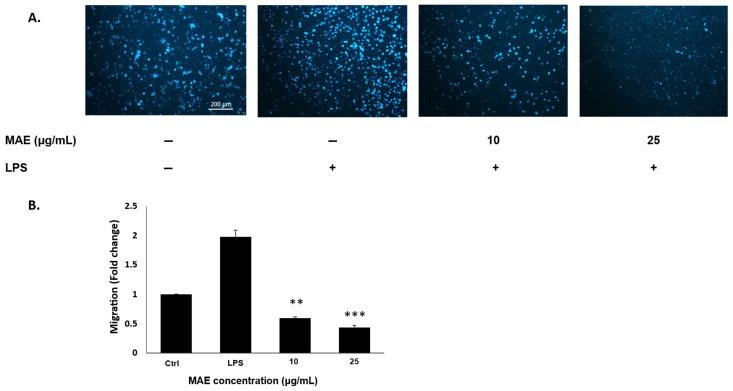
(**A**) RAW 264.7 cells were exposed to no treatment (negative control), LPS, or a combination of LPS and MAE at the specified concentrations for 24 h. The Trans well migration assay was used to assess cellular migration, and MAE significantly prevented LPS-induced migration of RAW 264.7 cells. Following DAPI (4′,6-diamidino-2-phenylindole) which is a popular blue fluorescent stain that binds strongly to A-T rich regions in double-stranded DNA, migratory cells were examined under a fluorescence microscope at 4x magnification. (**B**) Values are shown as the mean ± SEM of three separate experiments and are expressed as fold change relative to the control (** *p* < 0.01 and *** *p* < 0.001). Values of triplicates were presented in Table S4 in the Supplementary Materials.

The inflammatory response is maintained in chronic inflammation by the constant migration of macrophages to the afflicted tissues and the release of pro-inflammatory mediators [23]. MAE significantly decreased the migration potential of LPS-induced RAW 264.7 cells according to the Trans well migration assay results (Figure 4). MAE treatment significantly reduced the number of cells migrating from the upper to the lower chamber at both 10 and 25 µg/mL concentrations to 0.59 ± 0.01 and 0.43 ± 0.03-fold change, compared to an increase in the migration fold change by 1.97 ± 0.11 in the untreated LPS-induced cells.

### 2.5. MAE Inhibits the Activation of STAT-3 and COX-2 in RAW 264.7 Macrophages Activated by LPS

**Figure 5:**
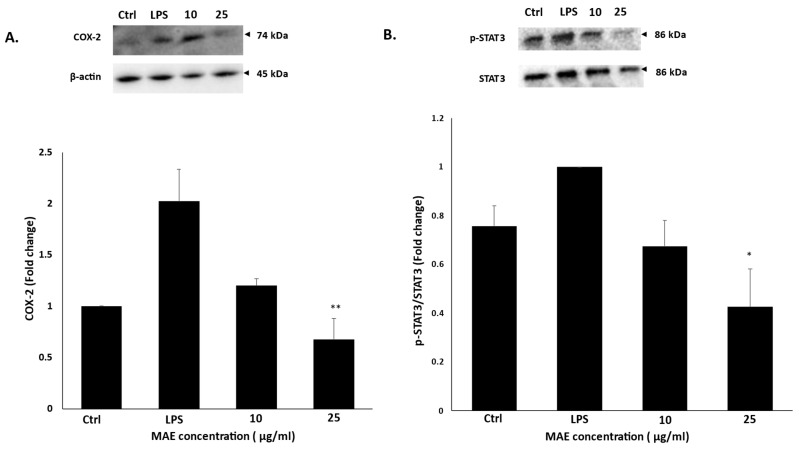
In LPS-induced RAW 264.7 cells, MAE suppresses COX-2 and STAT3 phosphorylation. For 24 h, RAW 264.7 cells were either left untreated (control), treated with LPS, or treated with a combination of LPS and MAE at the specified concentrations. Western blotting was used to assess the protein levels of COX-2 (**A**) and STAT3 (**B**). Values are shown as the mean ± SEM of three separate experiments and are expressed as the fold change relative to the control (** *p* < 0.01 and * *p* < 0.05). Values of triplicates were presented in Table S5 in the Supplementary Materials.

By mediating the transcription of pro-inflammatory genes like cytokines, NF-κB plays a crucial role in the LPS-induced inflammatory response, amplifying the immune response and promoting inflammation [24]. STAT-3 is a crucial regulator of inflammation, acting by promoting the expression of pro-inflammatory cytokines, such as TNF-α and IL-6, and facilitating the expression of inducible nitric oxide synthase (iNOS), which contributes to the inflammatory response [25]. 10 and 25 µg/mL MAE treatment decreased the phosphorylation levels of STAT-3 to 0.67 ± 0.1 and 0.42 ± 0.15 times, respectively, when compared to untreated LPS-induced cells. Moreover, a crucial enzyme in inflammation, COX-2, mainly mediates pain, swelling, and other inflammatory reactions by generating prostaglandins [26]. Our findings showed that COX-2 protein levels were down-regulated in LPS-induced cells treated with different concentrations of MAE for 24 h to 1.19 ± 0.06-fold and 0.67 ± 0.2-fold after treatment with 10 and 25 µg/mL of MAE, respectively, compared to control untreated cells (Figure 5).

### 2.6. MAE Inhibits the Phosphorylation of ERK, JNK, p38, and NF-κB in RAW 264.7 Macrophages

**Figure 6:**
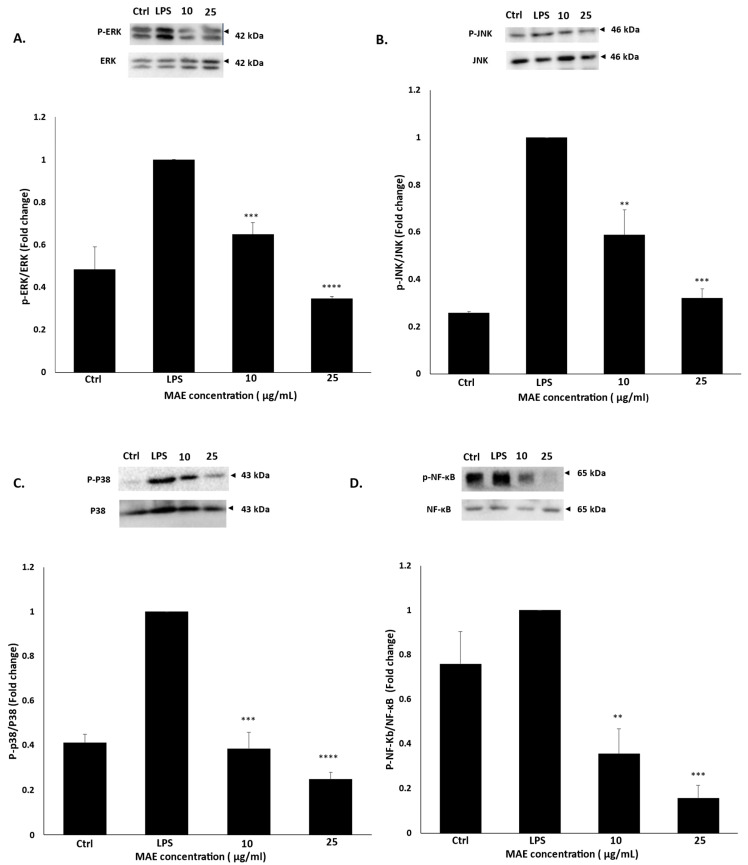
In RAW 264.7 cells stimulated with LPS, MAE prevents MAPK phosphorylation. RAW 264.7 cells were either left untreated (control), treated with LPS, or treated with a combination of MAE and LPS at the specified concentrations. Western blotting was used to assess the protein levels of (**A**) ERK1/2, (**B**) JNK, (**C**) P38, and (**D**) NF-κB. The values are shown as the mean ± SEM of three independent experiments and are expressed as the fold change relative to the control (** *p* < 0.01, *** *p* < 0.001 and **** *p* < 0.0001). Values of triplicates were presented in Table S6 in the Supplementary Materials.

Serine/threonine protein kinases, or MAPKs, are activated by a range of stimuli and regulate cellular responses [27]. In inflammation, ERK, JNK, and p38 specifically function upstream of NF-κB and are important signaling pathways that can trigger the NF-κB signaling pathway [28]. In this study, we aimed to ascertain the effect of MAE on NF-κB in LPS-induced RAW 264.7 cells. MAE treatment significantly decreased the phosphorylation of NF-ĸB in LPS-stimulated RAW 264.7 cells in a concentration-dependent manner; in fact, treatment with 10 and 25 µg/mL of MAE reduced the phosphorylation of NF-ĸB by 0.35 ± 0.1- and 0.15 ± 0.05-fold, respectively, when compared to the untreated LPS-induced cells. Thus, the effect of MAE against MAPKs was examined in LPS-induced RAW 264.7 cells. As shown in Figure 6, our findings demonstrate that treatment with MAE significantly reduces the activity of ERK, JNK, and p38, as indicated by a decrease in their phosphorylated forms. In fact, after administering a MAE concentration of 25 µg/mL to LPS-stimulated cells, the phosphorylation levels of ERK, JNK, and p38 dropped to 0.34 ± 0.009, 0.32 ± 0.03, and 0.24 ± 0.03, respectively, indicating that MAE suppresses inflammatory responses by blocking the MAPK signaling pathway.

### 2.7. MAE Stabilizes the Protein Denaturation of Casein and Bovine Albumin Serum

**Figure 7:**
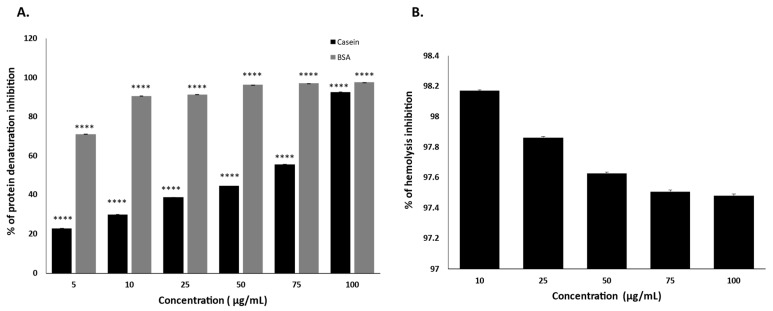
(**A**) The percentage of protein denaturation inhibition (BSA and casein) of increased concentrations of MAE. (**B**) The percentage of hemolysis inhibition of increased concentrations of MAE. Values are shown as the mean ± SEM of three separate experiments and are expressed as the fold change relative to the control (**** *p* < 0.0001). Percentages ± SEM were presented in Table S7 in the Supplementary Materials.

The inhibitory effect of the test sample on heat-induced protein denaturation was evaluated using casein and BSA as substrates at concentrations of 10, 25, 50, 75, and 100 µg/mL. In the casein denaturation assay, MAE exhibited a concentration-dependent increase in inhibition, with inhibition values of 30.11%, 55.63%, 38.89%, 44.68%, and 92.7% at concentrations of 5, 10, 25, 50, 75, and 100 µg/mL, respectively. Moderate inhibition was observed at low to intermediate concentrations, followed by a pronounced increase at the highest concentration tested. In the BSA denaturation assay, the test sample demonstrated strong inhibition across all tested concentrations. The percentage inhibition ranged from 90.5% at the lowest concentration to 97.56% at the highest concentration. MAE effectively inhibited heat-induced denaturation of both proteins, with consistently higher inhibition observed in the BSA model compared to casein (Figure 7A).

### 2.8. MAE Inhibits the Hemolysis of Heat-Induced Human Red Blood Cells

The anti-inflammatory activity of MAE was evaluated using the heat-induced red blood cell hemolysis assay. Exposure of erythrocytes to elevated temperature resulted in substantial hemolysis in the control group, whereas treatment with MAE markedly reduced hemoglobin release. MAE demonstrated strong inhibition of hemolysis across all tested concentrations. The percentage inhibition values were 98.17%, 97.86%, 97.63%, 97.51%, and 97.48% at concentrations of 10, 25, 50, 75, and 100 µg/mL, respectively. These results demonstrate that MAE effectively stabilizes erythrocyte membranes under thermal stress conditions (Figure 7B).

### 2.9. In Silico Molecular Docking with iNOS and ERK

The molecular docking analysis revealed variable binding affinities between the tested phytochemicals and the two inflammatory target proteins. Compounds with binding energies lower than −7 kcal/mol were considered to exhibit strong interactions [29]. Several compounds demonstrated strong binding affinity toward iNOS. The strongest interaction was observed with rutin (ΔG = −10.7 kcal/mol) followed by hyperoside (ΔG = −10.5 kcal/mol). The docking poses revealed the formation of multiple hydrogen bonds between hydroxyl groups of these flavonoid glycosides and amino acid residues located within the catalytic pocket of iNOS. Similarly, chrysin (ΔG = −9.6 kcal/mol) and quercetin (ΔG = −9.2 kcal/mol) exhibited strong binding interactions. Visualization of the docking complexes indicated hydrogen bond formation between the phenolic hydroxyl groups of these compounds and residues lining the active site of the enzyme.

Moderate but significant interactions were also observed for tropine (ΔG = −8.3 kcal/mol), chlorogenic acid (ΔG = −7.9 kcal/mol), and scopoletin (ΔG = −7.1 kcal/mol). These ligands formed one or more hydrogen bonds within the binding pocket, contributing to stabilization of the ligand–protein complex. Docking simulations with ERK revealed similar interaction trends. Quercetin (ΔG = −8.9 kcal/mol) showed the strongest predicted binding affinity, followed by chrysin (ΔG = −8.5 kcal/mol) and rutin (ΔG = −8.5 kcal/mol). The docking images demonstrated the formation of hydrogen bonds between these ligands and residues within the kinase binding pocket, suggesting stable interactions that may influence ERK activity. Additional compounds with notable binding affinity included hyperoside (ΔG = −7.9 kcal/mol) and chlorogenic acid (ΔG = −7.6 kcal/mol), which also displayed hydrogen bond interactions with amino acid residues in the protein cavity. The rest of the tested compounds showed less significant interactions, as shown in Table 1. The docking poses and interaction diagrams for the tested compounds are presented in Supplementary Figures S1 and S2.

## 3. Discussion

An essential biological reaction to damaging stimuli, such as infection and tissue damage, is inflammation [30]. When the immune system is unable to quickly eradicate the original cause of an infection or injury, chronic inflammation results [31]. Controlling inflammation is crucial for preventing chronic illnesses and preserving general health since it is linked to the onset and pathophysiology of numerous diseases [32]. Steroids and NSAIDs are effective anti-inflammatory medications, but prolonged use of these medications can have negative side effects [33]. NSAIDs produce anti-inflammatory, analgesic, and antipyretic effects primarily through inhibition of cyclooxygenase enzymes [34], reducing prostaglandin synthesis. While short courses are generally safe for most patients, prolonged or high-dose use is associated with important complications—especially gastrointestinal bleeding and ulceration, renal impairment (including acute kidney injury and sodium/water retention), and increased cardiovascular risk (hypertension, myocardial infarction, and heart failure exacerbation), the magnitude of which depends on the drug, dose, and patient comorbidities [35]. Likewise, long-term systemic glucocorticoid exposure is associated with dose- and duration-dependent harms including osteoporosis and fractures, metabolic disturbances (hyperglycemia, new-onset or worsened diabetes), increased susceptibility to infections, adrenal suppression, muscle weakness, cataracts, and neuropsychiatric effects [36]. These risks motivate the use of the lowest effective dose for the shortest necessary duration, the consideration of local/topical routes when feasible, prophylactic measures (e.g., bone-protective strategies or gastroprotection), and regular monitoring when chronic therapy is unavoidable [37]. As a result, using compounds derived from plants to create new anti-inflammatory drugs is gaining popularity [38]. For thousands of years, traditional medicine has utilized herbs with potent anti-inflammatory properties, including green tea, ginger, Boswellia, and turmeric [39]. These naturally occurring substances, which are often high in antioxidants, may reduce inflammation and offer a long-term, mild alternative to or addition to medications [40]. Numerous bioactive metabolites found in plants have been shown to have anti-inflammatory, anticancer, antioxidant, and antimicrobial [41]. Alkaloids, flavonoids, polyphenols, quinones, and tannins are some of these substances. As a result, current studies are still investigating the use of substances derived from plants as medicinal agents [42]. *Mandragora autumnalis*, sometimes referred to as the mandrake plant, witch’s herb, or devil’s herb, is one of these botanicals. It has long been regarded as one of the most significant medicinal plants and as an herb with significant cultural value. *Mandragora autumnalis* is an annual herb with thick rhizomes, oblong, ovate leaves, and blue-violet flowers [21]. *Mandragora autumnalis* is well known for its antioxidant and anticancer properties [16], but its anti-inflammatory activities have not yet been studied. Therefore, in the present study, we examined the anti-inflammatory activity of the ethanolic extract of *Mandragora autumnalis* in LPS-stimulated RAW 264.7 macrophages by evaluating its effects on the production of key pro-inflammatory cytokines and mediators showed a chemically varied profile made up of several classes of bioactive substances. The comprehensive phytochemical profiling of the same extract (MAE) was previously performed and published in a separate study. The extract used in the current manuscript is identical to that previously characterized, and its chemical composition was elucidated using LC–MS analysis in both positive and negative ionization modes. The LC–MS analysis of MAE revealed a chemically diverse profile comprising alkaloids, phenolic acids, flavonoids, fatty acids, and amide derivatives. In positive ionization mode, the most abundant compounds based on peak intensity were Hyoscyamine (m/z 290.1745; intensity ≈ 5.94 × 10^6^) and Hexadecanamide (Palmitic amide) (m/z 256.2629; intensity ≈ 7.81 × 10^6^), indicating that tropane alkaloids and fatty acid amides represent major constituents of the extract. Other notable alkaloids included Tropine, Tropinone, and Methylisopelletierine, which are characteristic metabolites of *Mandragora* species and may contribute significantly to the observed biological activities. Phenolic compounds were also clearly detected, including Chlorogenic acid (observed in both ionization modes with high intensity), Caffeic acid, and flavonoids such as Quercetin, Chrysin, Rutin, and Hyperoside. The presence of these phenolic and flavonoid compounds supports the potential antioxidant and anti-inflammatory properties of the extract [43]. Additionally, fatty acids and their derivatives, including Linoleic acid, Linolenic acid, Ethyl palmitate, and Ethyl linolenate, were identified, further contributing to the extract’s bioactive profile. In negative ionization mode, Chlorogenic acid, Scopoletin, Rutin, Hyperoside, and Linolenic acid were prominent, confirming the richness of MAE in phenolic and flavonoid glycosides [15]. The detection of these compounds in both ionization modes strengthens the reliability of compound identification Table 2 [15]. Overall, the LC–MS results demonstrate that MAE is a structurally well-characterized extract with clearly identified major and minor constituents.

Flavonoids have been demonstrated to mitigate oxidative stress and inflammation by reducing ROS, suppressing the NF-κB pathway, and lowering pro-inflammatory cytokines like TNF-α and IL-6 [44]. Two naturally occurring flavonoids with strong anti-inflammatory and immunomodulatory effects are quercetin and chrysin. Previous studies investigating the anti-inflammatory activity of chrysin in LPS-stimulated RAW 264.7 macrophages have typically used concentrations of 0, 1, 5, and 10 μM for 24 h [45]. Considering the molecular weight of chrysin (254.24 g/mol), these concentrations correspond approximately to 0, 0.25, 1.27, and 2.54 μg/mL, respectively. In our study, the crude *Mandragora autumnalis* extract (MAE) was tested at 10 and 25 μg/mL. Although the concentrations of the crude extract appear higher than those used for the pure compound, this difference is expected because MAE represents a complex mixture of phytochemicals, in which each compound is present only at a fraction of the total extract concentration. LC–MS analysis confirmed the presence of several bioactive constituents in MAE, including flavonoids such as quercetin and chrysin, as well as phenolic acids (e.g., chlorogenic acid and caffeic acid) and coumarins [15]. Therefore, the concentrations of MAE used in this study reflect the combined contribution of multiple active metabolites rather than a single purified compound [45]. On the other hand, it has been demonstrated that the therapeutic potential in autoimmune and chronic inflammatory conditions, such as rheumatoid arthritis, and its mechanisms involve the modulation of NF-κB and PI3K/Akt signaling pathways, which are crucial in inflammatory responses. Similar to this, chrysin (5,7-dihydroxyflavone), which is found in propolis, honey, and some plants, exhibits anti-inflammatory activity by suppressing the NLRP3 inflammasome, blocking IκB-α degradation, and inhibiting NF–κB activation. This lowers the production of pro-inflammatory mediators like NO, PGE_2_, COX–2, and different matrix-degrading enzymes. Chrysin demonstrated its immunomodulatory potential by reducing inflammatory cytokines, attenuating tissue damage, and increasing anti-inflammatory cytokines like IL-4 and IL-10 in preclinical models of osteoarthritis and autoimmune diseases [46]. Both quercetin and chrysin have poor bioavailability despite their encouraging effects, and current research is concentrated on creating better delivery methods or formulations to increase their therapeutic efficacy in inflammatory disorders [47].

It is well known that lipopolysaccharide (LPS), a crucial part of Gram-negative bacteria’s outer membrane, is a powerful inducer of immune activation [48]. It is frequently used to activate macrophages and trigger an inflammatory reaction in vitro [49]. LPS increases the expression and release of pro-inflammatory mediators and cytokines like nitric oxide (NO), inducible nitric oxide synthase (iNOS), cyclooxygenase-2 (COX-2), tumor necrosis factor-α (TNF-α), and different interleukins (ILs) after interacting with toll-like receptor 4 (TLR4) on the surface of macrophages [50,51]. Reactive oxygen species (ROS), which can worsen chronic inflammation by activating pro-inflammatory pathways like NF-κB, STAT-3, and MAPKs, are other significant signaling molecules that contribute to the development of inflammatory disorders. As a result, immune cells are drawn in, and cytokine production increases over time [52]. Oxidative stress generated by excessive production of reactive oxygen species (ROS) contributes to tissue injury and amplifies inflammatory signaling cascades [53]. Elevated ROS levels promote the activation of redox-sensitive transcription factors and inflammatory mediators, thereby sustaining chronic inflammation [53]. Numerous studies have demonstrated that pharmacological or natural antioxidant interventions that reduce ROS accumulation are effective in attenuating persistent inflammatory responses [54]. Pro-inflammatory cytokines such as tumor necrosis factor-α (TNF-α) and interleukin-6 (IL-6) play central roles in orchestrating inflammatory reactions, including leukocyte recruitment, vascular permeability, and amplification of downstream signaling networks [55].

In the present study, MAE significantly reduced TNF-α and IL-6 production in LPS-stimulated RAW 264.7 macrophages, suggesting potent cytokine-modulating and anti-inflammatory properties. Similar anti-inflammatory effects have been reported for several plant-derived extracts, which suppress macrophage activation by attenuating cytokine secretion and downstream signaling pathways [12]. The transcription factor NF-κB, a key regulator of inflammation, controls the expression of multiple pro-inflammatory genes, including iNOS, COX-2, and various cytokines [56]. NF-κB activation requires phosphorylation and proteasomal degradation of inhibitory IκB proteins, enabling nuclear translocation and transcriptional activation [57]. Upstream, this process is tightly regulated by major signaling cascades such as the mitogen-activated protein kinases (MAPKs), including p38, ERK1/2, and JNK, as well as the PI3K/Akt pathway, all of which interact with the NF-κB axis to drive inflammatory mediator production [58]. LPS engagement with Toll-like receptor 4 (TLR4) robustly activates these pathways in macrophages, triggering phosphorylation of MAPKs and initiating transcription of inflammatory genes [59]. Specifically, p38 MAPK regulates the expression of TNF-α and IL-6, ERK1/2 promotes cell survival and inflammatory gene expression, while JNK contributes to apoptosis and cytokine induction [60]. In this study, MAE treatment markedly reduced the phosphorylation of p38, ERK1/2, and JNK in LPS-activated RAW 264.7 cells, indicating effective suppression of MAPK pathway activation.

Among the identified compounds were chlorogenic acid, caffeic acid, quercetin, rutin, hyperoside, chrysin, scopoletin, hyoscyamine, tropinone, linoleic acid, and linolenic acid, in addition to several other minor metabolites. Notably, chlorogenic acid was detected with relatively high intensity in both ionization modes, suggesting that it represents one of the major phenolic constituents of the extract [61]. Chlorogenic acid and its derivatives are well known for their antioxidant, anti-inflammatory, and antimicrobial activities, particularly through the inhibition of inflammatory mediators such as NO, COX-2, and pro-inflammatory cytokines, as well as the modulation of MAPK and NF-κB signaling pathways [62]. These activities are consistent with the inhibitory effects observed in the present study on NO production, pro-inflammatory cytokines (TNF-α and IL-6), and COX-2 expression in LPS-stimulated RAW 264.7 cells. Additionally, several flavonoids, including quercetin, rutin, hyperoside, and chrysin, were identified. These compounds are widely reported to exert strong anti-inflammatory and antioxidant properties, mainly through suppression of NF-κB activation and MAPK phosphorylation [63], which aligns with our findings showing that MAE significantly inhibited the phosphorylation of ERK, JNK, and p38 MAPKs as well as NF-κB signaling. Flavonoids such as quercetin are also known to downregulate STAT3 activation and COX-2 expression [64], further supporting the molecular mechanisms observed in our Western blot analyses. The extract also contained coumarins (e.g., scopoletin) and phenolic acids (e.g., caffeic acid), which are reported to possess anti-inflammatory and antimicrobial activities, including the inhibition of oxidative stress and inflammatory mediator production [65]. Furthermore, tropane alkaloids, such as hyoscyamine and tropinone, were detected. Alkaloids from *Mandragora* species are known to contribute to the biological activity of the plant and may participate in the observed antimicrobial and anti-inflammatory effects [21]. Finally, the detection of unsaturated fatty acids, including linoleic and linolenic acids, may also contribute to the anti-inflammatory activity of the extract, as these compounds are reported to modulate inflammatory pathways and reduce cytokine production [66]. Taken together, the combined presence of phenolic acids, flavonoids, coumarins, alkaloids, and fatty acid derivatives likely acts synergistically to produce the biological activities observed in this study. The anti-inflammatory effects of MAE, particularly the inhibition of NO production, pro-inflammatory cytokines, COX-2 expression, and MAPK/NF-κB signaling pathways, are therefore strongly supported by the phytochemical profile revealed by the LC–MS analysis.

**Figure 8:**
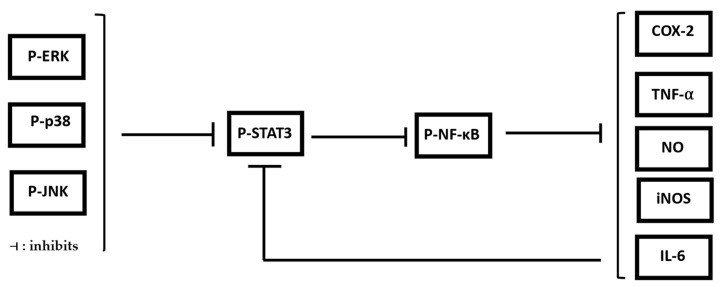
The proposed mechanism of action of MAE in the treatment of RAW 264.7 through the mediation of the selected markers (**⊣**: inhibits).

These findings align with previously reported anti-inflammatory mechanisms of multiple medicinal plant extracts, which mitigate inflammation by concomitantly inhibiting MAPK and NF-κB signaling pathways [67]. Collectively, these results demonstrate that MAE exerts a multi-targeted anti-inflammatory effect by reducing ROS-associated signaling, downregulating key cytokines, and suppressing MAPK/NF-κB pathway activation [68] (Figure 8).

Moreover, molecular docking was performed to evaluate the potential interaction of major phytochemical constituents identified in *Mandragora autumnalis* with two inflammation-related proteins: inducible nitric oxide synthase (iNOS) and extracellular signal-regulated kinase (ERK). These targets were selected due to their key roles in inflammatory signaling pathways, particularly the MAPK pathway and nitric-oxide-mediated inflammatory responses [69]. The molecular docking results suggest that several phytochemicals identified in *Mandragora autumnalis*, particularly flavonoids and phenolic compounds, exhibit strong predicted interactions with key inflammatory proteins such as iNOS and ERK. Flavonoids including rutin, hyperoside, quercetin, and chrysin demonstrated the strongest binding affinities toward both targets. These compounds possess multiple hydroxyl groups capable of forming hydrogen bonds with amino acid residues within protein binding pockets. Such interactions are known to significantly enhance ligand–protein stability and may contribute to inhibition of enzymatic activity [70]. The strong interaction with iNOS suggests that these compounds may interfere with nitric oxide production, which plays a central role in inflammatory processes [71]. Similarly, interaction with ERK may influence the MAPK signaling pathway, which regulates the expression of various inflammatory mediators [71]. Previous studies have reported that flavonoids such as quercetin and rutin can modulate both nitric oxide synthase activity and MAPK signaling pathways, supporting the docking predictions observed in this study [72]. The presence of hydrogen bond interactions observed in the docking models further supports the potential stability of these ligand–protein complexes [73]. However, it is important to note that the current study investigated a crude plant extract rather than isolated compounds, and therefore the observed biological effects may result from synergistic interactions among multiple phytochemicals. While molecular docking provides useful insights into possible mechanisms of action, experimental validation using purified compounds and enzyme inhibition assays is necessary to confirm these predicted interactions [74]. Overall, the docking findings support the hypothesis that polyphenolic constituents of *Mandragora autumnalis* may contribute to its biological activity through potential interaction with key inflammatory targets such as iNOS and ERK.

Apart from its impact on inflammatory mediators, MAE has also been shown to prevent inflammatory cells from migrating, which is a crucial step in the inflammatory response. The body’s defensive mechanism depends heavily on cell migration, which enables immune cells to reach areas of damage or infection [75]. Dysregulated or excessive cell migration can lead to chronic inflammation, contributing to various diseases, such as autoimmune disorders and cancer. MAE may help limit the spread of inflammation by preventing this migration, which would lessen the tissue damage that frequently results from inflammatory reactions [21]. MAE’s dual function in regulating inflammatory mediators and cell migration underscores its potential therapeutic advantages in the control of inflammation.

On the other hand, our present study evaluated the anti-inflammatory potential of the test sample using in vitro protein denaturation inhibition assays with both casein and bovine serum albumin (BSA) as model proteins. Protein denaturation assays are widely accepted as preliminary screening tools for anti-inflammatory activity because the stabilization of protein structure under stress conditions correlates with the ability to prevent inflammatory protein damage which was shown in a previous study that demonstrated the anti-inflammatory activity of *Sinapis arvensis* flower extract and Diclofenac which was assessed using the BSA protein denaturation assay at 25, 50, and 100 μg/mL. Both showed concentration-dependent inhibition, reaching 81.8% for the extract and 100% for Diclofenac at 100 μg/mL [76]. In our assays, BSA denaturation was strongly inhibited at all tested concentrations, with percent inhibition ranging from approximately 90.5% at the lowest concentration to over 97.5% at the highest concentration (100 µg/mL). This marked inhibition suggests a robust protective effect of the test sample against heat-induced protein unfolding, consistent with findings reported in similar studies where natural products and anti-inflammatory compounds effectively stabilized BSA against thermal denaturation. Such stabilization is interpreted as an ability to prevent the exposure of hydrophobic residues and aggregation that normally accompanies denaturation, processes implicated in eliciting inflammatory responses [76,77]. By contrast, the casein denaturation assay exhibited a more variable trend, with lower inhibitory effects at intermediate concentrations (38.9–55.6%) and a pronounced increase to 92.7% inhibition at the highest concentration. This pattern likely reflects intrinsic differences between the structural properties of casein and globular proteins like BSA. Casein exists as a complex micellar aggregate with multiple sub-structures that respond differently to thermal stress, which can lead to non-linear inhibition profiles in denaturation assays (e.g., variable exposure of reactive sites) [78]. Despite this variability, the high inhibition at 100 underscores the concentration-dependent capability of the test sample to exert protein stabilization, aligning with prior reports that powerful anti-inflammatory agents often show enhanced inhibition at higher doses in denaturation models [79]. The dose-dependent inhibition observed in both models is biologically meaningful, as inhibition of protein denaturation is hypothesized to reflect the capacity to protect tissue proteins against structural and functional disruptions during inflammation. Classical anti-inflammatory drugs such as nonsteroidal anti-inflammatory drugs (NSAIDs) have been shown to inhibit thermal denaturation of proteins like BSA, and such inhibition correlates with in vivo anti-inflammatory efficacy in animal models (e.g., carrageenan-induced edema) [80]. Although the protein denaturation assay does not directly measure cellular inflammatory mediators (e.g., cytokines, COX-2 activity), it provides a relevant biochemical surrogate for the anti-inflammatory potential of compounds at an early screening stage [81]. Overall, the consistently high inhibition in the BSA model, combined with the clear concentration dependence of casein stabilization at higher doses, supports the interpretation that the test sample possesses significant anti-inflammatory potential. This conclusion is strengthened by the well-established utility of BSA and other protein denaturation assays in screening phytochemicals and synthetic agents for anti-inflammatory properties, with higher percent inhibition generally indicating stronger activity. Furthermore, the heat-induced red blood cell hemolysis assay is a widely accepted in vitro model for evaluating anti-inflammatory activity based on membrane stabilization. During inflammatory processes, lysosomal membrane destabilization leads to the release of proteases and other inflammatory mediators that exacerbate tissue injury. Because erythrocyte membranes closely resemble lysosomal membranes in composition, protection against heat-induced hemolysis is considered a reliable indicator of potential anti-inflammatory effects, which was previously emphasized by a study that used Bergapten, a furocoumarin, and tested it at different concentrations, including 10, 30, and 100 μg/mL for protection against human erythrocyte hemolysis and protein denaturation. The extract exhibited significant concentration-dependent protection, confirming its anti-inflammatory potential [82]. In the present study, MAE exhibited remarkably high inhibition of heat-induced RBC hemolysis (>97%) at all concentrations tested, indicating strong membrane-stabilizing properties. Such potent inhibition suggests that MAE effectively protects membrane integrity against thermal stress, thereby preventing erythrocyte lysis and hemoglobin release. Comparable levels of hemolysis inhibition have been reported for established anti-inflammatory agents and plant-derived compounds rich in phenolics, flavonoids, and alkaloids [82]. Notably, the inhibitory effect of MAE showed minimal variation with increasing concentration, suggesting that effective membrane stabilization may occur at relatively low doses. This pattern has been previously reported and may reflect early saturation of membrane interaction sites or high affinity of active constituents for membrane phospholipids and proteins. The presence of bioactive secondary metabolites in *Mandragora autumnalis* may contribute to this effect by reinforcing membrane structure and reducing susceptibility to heat-induced rupture. Although the heat-induced hemolysis assay does not directly measure inflammatory mediators or intracellular signaling pathways, it provides valuable preliminary evidence of anti-inflammatory potential through a well-established biochemical mechanism. These findings complement other in vitro anti-inflammatory assays and support further investigation of MAE [77].

## 4. Materials and Methods

### 4.1. RAW 264.7 Macrophage Cell Culture

DMEM (Dulbecco’s Modified Eagle’s Medium) high glucose medium was used to cultivate RAW 264.7 macrophages (American Type Culture Collection, Manassas, VA, USA). 10% fetal bovine serum (FBS; Sigma-Aldrich, St. Louis, MO, USA) and 1% penicillin/streptomycin (Corning, MA, USA) were added as supplements. The cells were kept at 37 °C with 5% CO_2_ in a humidified incubator

### 4.2. Collection and Preparation of the Ethanolic Extract of Mandragora autumnalis

The leaves of *Mandragora autumnalis* were cleaned and then left to air dry at room temperature. The plants were recognized by Mohammad Al-Zein, a resident plant taxonomist at the American University of Beirut (AUB) herbarium, with the identification number GA 2025-1. They were then finely ground into a powder and left in 80% ethanol for 72 h. After passing through filter paper, the suspension was subjected to a rotary evaporator to evaporate ethanol, then lyophilized to remove water using a freeze-dryer. The resultant residue was kept at 4 °C in the dark after being dissolved in DMSO at a concentration of 100 mg/mL. A stock solution of the plant extract was prepared by dissolving 500 mg of the extract in 500 mL of DMSO (A fresh stock solution of the plant extract was prepared every 24 h to ensure stability and reproducibility of the experiments, and the desired working concentrations were then obtained by diluting the stock in DMEM high-glucose medium).

### 4.3. Cell Viability MTT Assay

RAW 264.7 cells were seeded at a density of 10,000 cells per well in a 96-well tissue-culture plate and allowed to grow until they reached 40% confluence. The cells were then treated with increasing concentrations (10, 25, 50, 75, and 100 µg/mL) of MAE for 24 h. These doses cover a biologically relevant range, allowing assessment of dose-dependent activity while remaining below cytotoxic levels reported in the literature [83]. Cell viability was assessed using the reduction of 3-(4,5-dimethylthiazol 2-yl)-2,5-diphenyltetrazolium bromide (MTT; Sigma-Aldrich). Viability was determined by comparing the treated cells to vehicle-treated cells (equivalent concentration of DMSO). The absorbance was measured at 595 nm using an ELISA microplate reader (Thermo Scientific MULTISKAN GO, Waltham, MA, USA).

### 4.4. Western Blot Analysis and Whole-Cell Protein Extract Preparation

RAW 264.7 cells were lysed in a lysis buffer containing 60 mM Tris and 2% SDS (pH of 6.8) after two PBS washes to produce whole-cell lysates. Protein extracts were separated using 10% SDS-PAGE and then transferred to a polyvinylidene difluoride (PVDF) membrane in aliquots of 25–30 µg (Immobilon PVDF; Bio-Rad, Hongkong, China). 5% non-fat dry milk powder in TBST (Tris-buffered saline with 0.05% Tween 20) was used to block the PVDF membrane for one hour at room temperature. The membrane was incubated overnight with particular primary antibodies at 4 °C. Then the membrane was incubated with a horseradish peroxidase-conjugated anti-IgG secondary antibody for two hours following the removal of the primary antibody and TBST washing. After that, TBST was used to remove the secondary antibodies. The immunoreactive bands were seen using an enhanced chemiluminescence (ECL) substrate kit (Bio Rad). Densitometry analysis was used to quantify the Western blot bands, and to guarantee accuracy and consistency, normalization to β-actin was applied. All primary and secondary antibodies were obtained from Cell Signaling (Cell Signaling Technology, Inc., Danvers, MA, USA) [24,84].

### 4.5. RNA Extraction for the Quantitative Real-Time PCR Analysis

Trizol reagent (Sigma-Aldrich) was used to extract total RNA in accordance with the manufacturer’s instructions. A Nanodrop 2000 spectrophotometer (Thermo Scientific, Waltham, MA, USA) was used to measure absorbance at 260 nm in order to determine the concentration of RNA. The ReadyScript cDNA Synthesis Mix (Sigma-Aldrich) was utilized to synthesize cDNA from one microgram of RNA from each sample. The iTaq universal SYBR Green Supermix (Sigma-Aldrich) and the CFX96 PCR-detection system (Bio-Rad) were used to perform quantitative real-time PCR (qPCR). Table 3 lists the precise primer sequences that were used. 40 cycles of denaturation at 95 °C for 30 s and annealing/extension at 60 °C for 1 min comprised the qPCR conditions, which also included an initial activation step at 95 °C for 3 min [24].

### 4.6. Nitric Oxide Production Measurement

The Griess Reagent Nitrite Measurement Kit (Cell Signaling Technology, Inc., Danvers, MA, USA) was used to measure the concentration of nitrite in order to quantify the levels of nitric oxide (NO). As directed by the manufacturer, sulfanilic acid and naphthylethylenediamine dihydrochloride solutions were combined to create the Griess Reagent. For a duration of 24 h, RAW 264.7 cells (2.5 × 10^5^ cells/mL) were given either no treatment, LPS, or a combination of LPS and MAE at concentrations of 10 and 25 µg/mL. The prepared Griess Reagent was added to each sample in an equal volume and thoroughly mixed. After 15 min of letting the reaction develop at room temperature, absorbance at 550 nm was measured. The absorbance values of the samples were compared to a standard curve created from nitrite standards of known concentrations to ascertain the nitrite concentrations. To ensure accuracy, every measurement was carried out three times [24].

### 4.7. Cell Migration Evaluation Using the Trans Well Assay

Trans well inserts with an 8-μm pore size (BD Biosciences, Bedford, MA, USA) were used to evaluate the migratory potential of RAW 264.7 cells. For a duration of 24 h, RAW 264.7 cells (2.5 × 10^5^ cells/mL) were given either no treatment, LPS, or a combination of LPS and MAE at concentrations of 10 and 25 µg/mL. After that, the cells were transferred to the upper chamber of the insert. As a chemoattractant, 10% fetal bovine serum was added to the DMEM in the lower wells. A sterile cotton swab was used to remove non-migratory cells from the filter’s upper surface following a three-hour incubation period at 37 °C. After successfully migrating to the lower surface, the cells were stained with DAPI, fixed with 4% formaldehyde, and examined under a fluorescence microscope. Three replicates of the experiment were conducted, and the results were displayed as mean values ± SEM [15].

### 4.8. Heat-Induced Hemolysis of Red Blood Cells

100 µL of MAE at concentrations of 10, 25, 50, 75, and 100 μg/mL in isotonic PBS and 100 µL of a 10% RBC suspension made up the assay mixture. After centrifuging the reaction mixtures for five minutes at 3000 rpm and incubating them for twenty minutes at 54 °C in a water bath (for the heated samples) and at 37 °C for the non-heated samples, the absorbance of the supernatant was measured at 540 nm using a spectrophotometer [81]. The following formula was used to determine the percentage inhibition of hemolysis:

$$\%\mathrm{Inhibition}\;\mathrm{of}\;\mathrm{Hemolysis}\;=\;100\;\times \;1\;-\;\frac{\left(\mathrm{ODheated}\;\mathrm{sample}\;-\;\mathrm{ODunheated}\;\mathrm{sample}\right)}{\left(\mathrm{ODheated}\;\mathrm{sample}\;-\;\mathrm{ODunheated}\;\mathrm{control}\right)}$$

### 4.9. Protein Denaturation Assay

A 1 mL reaction mixture comprising 500 µL of 5% bovine albumin serum or 1% casein, 400 µL of phosphate-buffered saline (PBS, pH 6.4), and 100 µL of MAE at varying concentrations (10, 25, 50, 75, and 100 μg/mL) in isotonic PBS. As a control, isotonic PBS of comparable volume was utilized. The reaction mixtures were heated to 70 °C for thirty minutes. After cooling, the absorbance was measured at 660 nm using a spectrophotometer. The equation was used to calculate the inhibition percentage of protein denaturation [81].

$$\%\mathrm{Inhibition}\;\mathrm{of}\;\mathrm{Protein}\;\mathrm{Denaturation}=100\;\times \;1\;-\;\frac{\left(\mathrm{Turbidity}\;\mathrm{of}\;\mathrm{tested}\;\mathrm{sample}\;-\;\mathrm{Turbidity}\;\mathrm{of}\;\mathrm{absence}\;\mathrm{of}\;\mathrm{sample}\right)}{\mathrm{Turbidity}\;\mathrm{of}\;\mathrm{tested}\;\mathrm{sample}}$$

### 4.10. In Silico Molecular Docking

An in silico molecular docking analysis was performed to investigate the potential interactions between selected ligands, and target proteins involved in cellular signaling pathways using the Mcule molecular docking platform (https://mcule.com/). The prepared ligands were docked into the active sites of iNOS and ERK to evaluate their potential binding affinity and interaction patterns. The docking poses were visualized and analyzed to identify key intermolecular interactions, particularly hydrogen bonds between ligand functional groups and amino acid residues within the protein binding pocket using Mcule. For the ligand preparation, the chemical structures of the phytochemical compounds identified in *Mandragora autumnalis* were obtained from the PubChem database in three-dimensional (3D) format. The selected compounds include flavonoids, phenolic acids, alkaloids, and fatty acid derivatives such tropinone, methylisopelletierine, solacaproine, quercetin, tropine, chrysin, rutin, hyperoside, chlorogenic acid, caffeic acid, scopoletin, linoleic acid, hexadecanamide (palmatic amide) were obtained from the PubChem database (https://pubchem.ncbi.nlm.nih.gov/). For the protein preparation, the three-dimensional crystal structures of the target proteins inducible nitric oxide synthase (iNOS) and extracellular signal-regulated kinase (ERK) corresponding to Protein Data Bank entries 2y37 and 3R1N, respectively, were retrieved from the Protein Data Bank (PDB; https://www.rcsb.org/). The prepared ligands were docked into the active sites of iNOS and ERK to evaluate their potential binding affinity and interaction patterns. Binding energies were calculated as Gibbs free energy changes (ΔG, kcal/mol), where more negative values indicate stronger predicted ligand–protein interactions [29]. The docking poses were visualized and analyzed in Mcule to identify key intermolecular interactions, particularly hydrogen bonds between ligand functional groups and amino acid residues within the protein binding pocket.

### 4.11. Statistical Analysis

All experimental data from the three separate experiments were presented as the mean ± standard error of the mean (SEM). Dunnett’s post hoc test was used to compare the treated groups with the control group after one-way analysis of variance (ANOVA) was completed. Statistical significance was defined as a *p*-value of less than 0.05.

## 5. Conclusions and Limitations

The findings of this study demonstrated that *Mandragora autumnalis* ethanolic extract (MAE) exhibits significant anti-inflammatory activity in LPS-stimulated RAW 264.7 macrophages. MAE reduced nitric oxide production and pro-inflammatory cytokine secretion (TNF-α and IL-6) through the downregulation of iNOS and COX-2 expression. In addition, MAE inhibited macrophage migration and suppressed key inflammatory signaling pathways, including NF-κB, STAT3, and MAPKs, indicating broad regulatory effects on inflammatory processes. MAE also demonstrated strong in vitro anti-inflammatory potential through its ability to inhibit heat-induced protein denaturation and stabilize red blood cell membranes, suggesting a protective role against inflammatory membrane damage. These biological effects may be attributed to the diverse phytochemical composition of the extract and possible synergistic interactions among its bioactive constituents. Nevertheless, further studies are required to isolate and characterize the active compounds, investigate structure–activity relationships, and validate these effects in vivo. Additional research addressing pharmacokinetics, safety, and potential toxicity will be essential to evaluate the therapeutic potential of MAE as a natural anti-inflammatory agent. One limitation of this study is that the molecular docking analysis was performed only on selected proteins and a limited number of major chemical compounds identified in the extract. Therefore, a comprehensive molecular docking investigation including all identified compounds against a broader panel of relevant target proteins should be conducted in future studies to better elucidate the full spectrum of potential molecular interactions and mechanisms of action.

## Figures and Tables

**Table 1:** Molecular docking binding energies (ΔG) of chemical compounds with ERK and iNOS.

Ligand	ΔG (kcal/mol) with iNOS	ΔG (kcal/mol) with ERK
Chloronergic acid	−7.9	−7.6
Tropine	−8.3	−7.1
Quercetin	−9.2	−8.9
Methylisopelletierine	−5	−4.7
Tropinone	−4.2	−4.4
Solacaproine	−5.9	−5.2
Chrysin	−9.6	−8.5
Rutin	−10.7	−8.5
Hyperoside	−10.5	−7.9
Caffeic acid	−6.8	−6.6
Scopoletin	−7.1	−6.6
Linoleic acid	−5.9	−5.4
Hexadecanamide	−5.5	−5.2

**Table 2:** LC-MS analysis of MAE with positive and negative ionization modes [15].

A. Positive Ionization Mode						
Number	m/z	RT [min]	Ions	Compound Name	Molecular Formula	Intensity
1	127.0389	0.58	[M + H]+	5-Hydroxymethyl-2-furancarboxaldehyde	C_6_H_6_O_3_	49,130.136
2	133.0827	0.71	[M + H]+	Ethyl 3-hydroxy-butanoate	C_6_H_12_O_3_	72,955.974
3	140.1066	0.86	[M + H]+	Tropinone	C_8_H_13_NO	21,053.859
4	149.0596	1.23	[M + H]+	3-(Methylthio)propyl acetate	C_6_H_12_O_2_S	8790.512
5	117.0542	1.3	[M + H]+	1-Hydroxy-2-propanone acetate	C_5_H_8_O_3_	8646.324
6	193.0492	2.91	[M + H-C6H10O5]+	Chlorogenic acid	C_16_H_18_O_9_	669,015.883
	355.1018		[M + H]+			221,652.966
	445.0708		[M + Na + NaCOOH]+			20,681.809
7	619.2479	2.91	[M + H]+	Simulanoquinoline	C_37_H_34_N_2_O_7_	11,326.110
8	641.2302		[M + Na]+			90,245.22
9	290.1745	3.89	[M + H]+	Hyoscyamine	C_17_H_23_NO_3_	5,938,808.003
10	303.0494	9.16	[M + H]+	Quercetin	C_15_H_10_O_7_	20,868.344
11	179.1178	9.6	[M + H]+	Ethyl hydrocinnamate	C_11_H_14_O_2_	24,858.923
12	255.0862	13.43	[M + H]+	Chrysin	C_15_H_10_O_4_	11,043.2
13	281.266	26.76	[M + H]+	Linoleic acid	C_18_H_32_O_2_	70,427.883
14	311.2933	27.7	[M + H]+	Ethyl oleate	C_20_H_38_O_2_	4151.762
15	243.2505	28.62	[M + H]+	n-Pentadecanoic acid	C_15_H_30_O_2_	13,652.643
16	193.1581	29.04	[M + H]+	Ionone (β-Ionone)	C_13_H_20_O	10,667.398
17	307.266	29.32	[M + H]+	Ethyl linolenate	C_20_H_34_O_2_	10,224.308
18	156.138	29.41	[M + H]+	Methylisopelletierine	C_9_H_17_NO	48,479.510
19	114.0911	29.41	[M + H-C2H4]+	Tropine	C_8_H_15_NO	42,742.76
	142.1224		[M + H]+			57,239.581
20	336.2868	29.49	[M + Na]+	Solacaproine	C_18_H_39_N_3_O	8765.329
21	256.2629	29.51	[M + H]+	Hexadecanamide (Palmitic amide)	C_16_H_33_NO	7,806,388.659
	278.2449		[M + Na]+			1,763,628.958
	511.5185	29.52	[2M + H]+			449,241.592
	533.5006		[2M + Na]+			363,277.043
	294.2182		[M + K]+			35,062.909
22	297.2893		[M + H-NH3]+	Solacaproine	C_18_H_39_N_3_O	86,740.906
	314.3049		[M + H]+			52,611.286
23	285.2879	29.77	[M + H]+	Ethyl palmitate	C_18_H_36_O_2_	58,073.530
B. Negative ionization mode						
24	111.0088	0.75	[M-H]-	3-Methyl-2-5-furandione	C_5_H_4_O_3_	100.285
25	117.01932	0.83	[M-H]-	Succinic acid	C_4_H_6_O_4_	12,012
26	353.08783	2.21	[M-H]-	Chlorogenic acid	C_16_H_18_O_9_	258,912
27	207.050913	3.29	[M-H]-	4-O-Methylglucuronic acid	C_7_H_12_O_7_	4598.659
28	131.07127	3.33	[M-H]-	Ethyl 3-hydroxy-butanoate	C_6_H_12_O_3_	2582
29	179.03492	3.86	[M-H]-	Caffeic Acid	C_9_H_8_O_4_	5934
30	175.04000	4.23	[M-H-COCH2]-	4-Methylumbelliferyl acetate	C_12_H_10_O_4_	27,106.992
	217.05106		[M-H]-			4492.950
31	176.01133	6.56	[M-H-CH3]-	Scopoletin	C_10_H_8_O_4_	26,388.932
	191.03486		[M-H]-			43,250.386
	259.02198		[M-H + NaCOOH]-			9135.296
32	609.1457	9.19	[M-H]-	Rutin	C_27_H_30_O_16_	25,850
33	463.08799	10.39	[M-H]-	Hyperoside	C_21_H_20_O_12_	18,198
34	277.21675	29.84	[M-H]-	Linolenic acid	C_18_H_30_O_2_	51,774.794
	345.20465		[M-H + NaCOOH]-			4077.093

**Table 3:** Sequences of primers used for qRT-PCR.

Genes	Forward	Reverse
*TNF* *α*	G T A G C C C A C G T C G T A G C A A A C C A C	G G T A C A A C C C A T C G G C T G G C A C
*IL-6*	C C T C T C T G C A A G A G A C T T C C A T C C A	T C C T C T G T G A A G T C T C C T C T C C G G
*COX2*	G A T A C T C A G G C A G A G A T G A T C T A C C C	A G A C C A G G C A C C A G A C C A A A G A

## Data Availability

The original contributions presented in this study are included in the article and Supplementary Materials. Further inquiries can be directed to the corresponding authors.

## Supplementary Materials

The following supporting information can be downloaded at: https://www.mdpi.com/article/10.3390/ph19030483/s1, Figure S1: Molecular docking conformations of selected chemical compounds within the active site of inducible nitric oxide synthase (iNOS); Figure S2: Molecular docking conformations of selected chemical compounds within the active site of ERK. Table S1: MTT assay results showing average cell viability (%) after 24-h treatment with MAE at indicated concentrations (5, 10, 25, 50, 75 and 100 μg/mL); data are mean ± SD of three independent replicates compared to vehicle control; Table S2: Effect of MAE on nitric oxide production and iNOS protein expression in LPS-stimulated RAW 264.7 cells. RAW 264.7 cells were left untreated (control), stimulated with LPS, or co-treated with MAE (10 and 25 μg/mL) and LPS for 24 h. (A) iNOS protein expression was evaluated by Western blot analysis, and (B) nitrite levels were determined using the Griess reagent assay. Data are presented as mean ± SEM from three independent experiments performed in triplicate and expressed as fold change relative to the control; Table S3: Effect of MAE on the mRNA expression of pro-inflammatory mediators in LPS-stimulated RAW 264.7 cells. RAW 264.7 cells were left untreated (control), stimulated with LPS, or co-treated with LPS and MAE at the indicated concentrations for 24 h. The mRNA expression levels of TNF-α (A), IL-6 (B), and COX-2 (C) were determined by RT-PCR. Data are presented as mean ± SEM from three independent experiments performed in triplicate and expressed as fold change relative to the control; Table S4: RAW 264.7 cells were left untreated (negative control), stimulated with LPS, or co-treated with LPS and MAE at the indicated concentrations for 24 h. Cell migration was evaluated using a Trans-well migration assay, and migratory cells were visualized after DAPI staining under a fluorescence microscope. Data are presented as mean ± SEM from three independent experiments performed in triplicate and expressed as fold change relative to the control; Table S5: Effect of MAE on COX-2 and STAT3 protein expression in LPS-stimulated RAW 264.7 cells. RAW 264.7 cells were left untreated (control), stimulated with LPS, or co-treated with LPS and MAE at the indicated concentrations for 24 h. Protein expression levels of COX-2 and phosphorylated STAT3 were determined by Western blot analysis. Data are presented as mean ± SEM from three independent experiments performed in triplicate and expressed as fold change relative to the control; Table S6: Effect of MAE on MAPK and NF-κB signaling pathways in LPS-stimulated RAW 264.7 cells. RAW 264.7 cells were left untreated (control), stimulated with LPS, or co-treated with LPS and MAE at the indicated concentrations for 24 h. Protein expression levels of ERK1/2, JNK, p38 MAPK, and NF-κB were evaluated by Western blot analysis. Data are presented as mean ± SEM from three independent experiments performed in triplicate and expressed as fold change relative to the control; Table S7: Effect of MAE on protein denaturation and hemolysis inhibition. Increasing concentrations of MAE were evaluated for their ability to inhibit hemolysis and protein denaturation (BSA and casein assays). Data are presented as percentages ± SEM.
