# Supplementary material for: Anti-Inflammatory Activity of Mandragora autumnalis Ethanolic Extract: In Vitro and Cellular Mechanistic Insights
Source: Pharmaceuticals (Basel). 2026 Mar 15;19(3):483. doi: 10.3390/ph19030483 (PMC13029175; doi:10.3390/ph19030483)
Supplement: Supplementary file 1 [file pharmaceuticals-19-00483-s001.zip › pharmaceuticals-4187036-supplementary.pdf]

Table S1. MTT assay results showing average cell viability (%) after 24-hour treatment with MAE at indicated concentrations (5, 10, 25, 50, 75 and 100 μg/mL); data are mean ± SD of three independent replicates compared to vehicle control.

	**Vehicle ctrl**	**5**	**10**	**25**	**50**	**75**	**100**
**n 1**	100	99.17658	94.00732	93.32113	97.52973	97.22476	84.53797
**n 2**	100	103.5734	98.69892	88.16199	86.34781	88.08869	101.521
**n 3**	100	93.86617	104.461	110.223	85.50186	93.86617	81.04089
**Avg**	100	98.87205	99.05573	97.23539	89.79313	93.05988	89.03328
**Stdv**	0	4.86077	5.23595	11.53965	6.71343	4.621096	10.95511
**Sem**	0	2.806367	3.022977	6.662422	3.876001	2.667991	6.324935

Table S2. Effect of MAE on nitric oxide production and iNOS protein expression in LPS-stimulated RAW 264.7 cells. RAW 264.7 cells were left untreated (control), stimulated with LPS, or co-treated with MAE (10 and 25 μg/mL) and LPS for 24 h. (A) iNOS protein expression was evaluated by Western blot analysis, and (B) nitrite levels were determined using the Griess reagent assay. Data are presented as mean ± SEM from three independent experiments performed in triplicate and expressed as fold change relative to the control.

**(A)**	**Ctrl**	**LPS**	**10**	**25**
**n1**	0.898287	1	0.702029	0.637721
**n2**	0.742488	1	0.724239	0.61853
**n3**	0.770768	1	0.938189	0.801757
**Avg**	0.803848	1	0.788152	0.686003
**Stdv**	0.083	0	0.130409	0.100704
**Sem**	0.04792	0	0.075292	0.058142

**(B)**	**Ctrl**	**LPS**	**10**	**25**
**n1**	0.042	0.065	0.054	0.055
**n2**	0.044	0.062	0.050	0.049
**n3**	0.041	0.062	0.047	0.046
**Avg**	0.042333	0.063	0.050333	0.050
**Stdv**	0.001528	0.001732	0.003512	0.004583
**Sem**	0.00108	0.001225	0.002483	0.00324

Table S3. Effect of MAE on the mRNA expression of pro-inflammatory mediators in LPS-stimulated RAW 264.7 cells. RAW 264.7 cells were left untreated (control), stimulated with LPS, or co-treated with LPS and MAE at the indicated concentrations for 24 h. The mRNA expression levels of TNF-α (A), IL-6 (B), and COX-2 (C) were determined by RT-PCR. Data are presented as mean ± SEM from three independent experiments performed in triplicate and expressed as fold change relative to the control.

(A)	Sample	Exp Fold Change	Stdv	Sem
TNF-α avg	Ctrl	1	0	0
	LPS	6.92670376	3.086093917	1.78175715
	10	2.018648722	1.749419741	1.01002796
	25	2.007671812	1.632809534	0.94270302

(B)	Sample	Exp Fold Change	Stdv	Sem
IL-6 avg	Ctrl	1	0	0
	LPS	29.19766788	3.47055767	2.0037274
	10	15.05869087	10.67828979	6.16511348
	25	9.702405713	3.876871718	2.23831293

(C)	Sample	Exp Fold Change	Stdv	Sem
COX-2 avg	Ctrl	1	0	0
	LPS	9.673499539	4.702501378	2.71499044
	10	4.737417546	4.682593553	2.70349665
	25	3.281179037	2.112788147	1.21981881

Table S4. RAW 264.7 cells were left untreated (negative control), stimulated with LPS, or co-treated with LPS and MAE at the indicated concentrations for 24 h. Cell migration was evaluated using a Trans-well migration assay, and migratory cells were visualized after DAPI staining under a fluorescence microscope. Data are presented as mean ± SEM from three independent experiments performed in triplicate and expressed as fold change relative to the control

	n1	n2	n3	Avg	Stdv	Sem
Ctrl	1	1	1	1	0	0
LPS	1.785714	2.047244	2.097625	1.976861	0.167444	0.118401
10	0.623016	0.566929	0.593668	0.594538	0.028053	0.019837

**25**	0.468254	0.456693	0.375989	0.433645	0.050265	0.035543

Table S5. Effect of MAE on COX-2 and STAT3 protein expression in LPS-stimulated RAW 264.7 cells. RAW 264.7 cells were left untreated (control), stimulated with LPS, or co-treated with LPS and MAE at the indicated concentrations for 24 h. Protein expression levels of COX-2 and phosphorylated STAT3 were determined by Western blot analysis. Data are presented as mean ± SEM from three independent experiments performed in triplicate and expressed as fold change relative to the control.

COX-2	Ctrl	LPS	10	25
**n1**	0.692749	1	0.190555	0.165276
**n2**	0.625937	1	0.375455	0.36243
**n3**	0.951437	1	1.011438	0.632884
**avg**	0.756707	1	0.525816	0.386863
**Stdv**	0.171917	0	0.430603	0.23476
**Sem**	0.099257	0	0.248609	0.135539

p-STAT3/STAT3	Ctrl	LPS	10	25
**n1**	0.781118	1	0.529248	0.127484
**n2**	0.600522	1	0.615644	0.501948
**n3**	0.889881	1	0.879719	0.648029
**avg**	0.757173	1	0.67487	0.42582
**stdv**	0.146158	0	0.182588	0.268493
**sem**	0.084384	0	0.105417	0.155014

Table S6. Effect of MAE on MAPK and NF-κB signaling pathways in LPS-stimulated RAW 264.7 cells. RAW 264.7 cells were left untreated (control), stimulated with LPS, or co-treated with LPS and MAE at the indicated concentrations for 24 h. Protein expression levels of ERK1/2, JNK, p38 MAPK, and NF-κB were evaluated by Western blot analysis. Data are presented as mean ± SEM from three independent experiments performed in triplicate and expressed as fold change relative to the control.

p-ERK/ERK	Ctrl	LPS	10	25
**n1**	0.690316	1	0.561613	0.32909
**n2**	0.41333	1	0.628505	0.358869

**n3**	0.344933	1	0.753019	0.352284
**avg**	0.48286	1	0.647712	0.346748
**stdv**	0.182889	0	0.097138	0.015643
**sem**	0.105591	0	0.056083	0.009031

p-JNK/JNK	Ctrl	LPS	10	25
**n1**	0.250419	1	0.376415	0.267331
**n2**	0.267875	1	0.686569	0.394733
**n3**	0.254612	1	0.700327	0.30386
**avg**	0.257635	1	0.58777	0.321975
**stdv**	0.009112	0	0.183168	0.065604
**sem**	0.005261	0	0.105752	0.037877

P-p38/p38	Ctrl	LPS	10	25
**n1**	0.432576	1	0.251778	0.201037
**n2**	0.33693	1	0.499803	0.233731
**n3**	0.461121	1	0.404535	0.308617
**avg**	0.410209	1	0.385372	0.247795
**stdv**	0.065047	0	0.125118	0.055152
**sem**	0.037555	0	0.072237	0.031842

P-NF-κB/NF-κB	Ctrl	LPS	10	25
**n1**	0.485257	1	0.268858	0.156806
**n2**	0.977844	1	0.573668	0.253759
**n3**	0.813335	1	0.222206	0.063441
**avg**	0.758812	1	0.354911	0.158002
**stdv**	0.250779	0	0.19088	0.095164
**sem**	0.144787	0	0.110205	0.054943

Table S7. Effect of MAE on protein denaturation and hemolysis inhibition. Increasing concentrations of MAE were evaluated for their ability to inhibit hemolysis and protein denaturation (BSA and casein assays). Data are presented as percentages ± SEM.

**Concentration (μg/mL)**	**5**	**10**	**25**	**50**	**75**	**100**
**% inhibition of Hemolysis**	98.4	98.17073	97.86423	97.62816	97.50862	97.48092
**Stdv**	0.008	0.009	0.0165	0.019	0.0185	0.003
**Sem**	0.0046188	0.00519615	0.00952628	0.01096966	0.01068098	0.00173205
**% inhibition of Casein denaturation**	23.0137	30.10753	38.88889	44.68085	55.62914	92.7
**Stdv**	0.004	0.001	0.001	0.001	0.001	0.001
**Sem**	0.002	0.001	0.000	0.001	0.001	0.000
**% inhibition of BSA denaturation**	71.14094	90.59829	91.37931	96.36364	97.08738	97.56098
**Stdv**	0.005	0.001	0.001	0.001	0.000	0.001
**Sem**	0.003	0.000	0.000	0.000	0.000	0.000

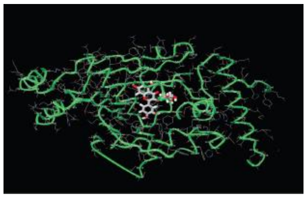

Hyperoside

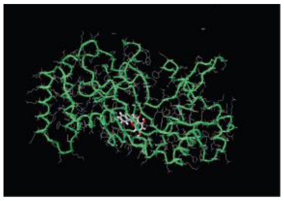

Chrysin

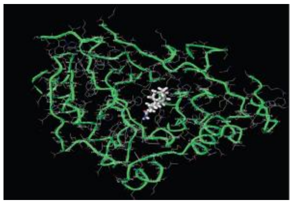

Hexadecanamide

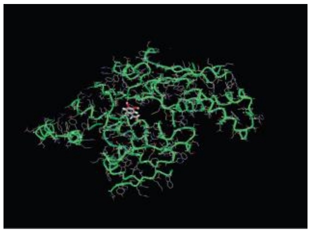

caffeic acid

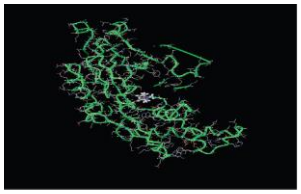

Tropinone

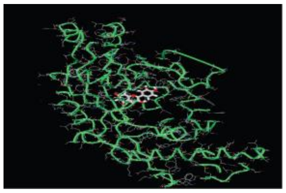

Quercetin

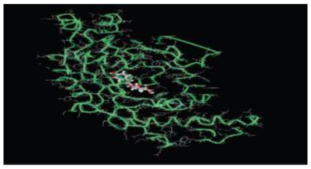

Chlorogenic acid

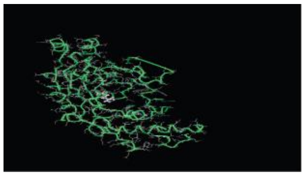

Methylisopelletierine

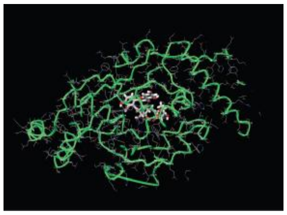

Rutin

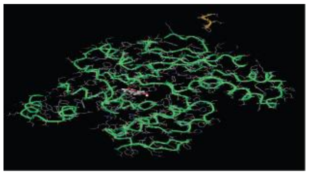

Scopoletin

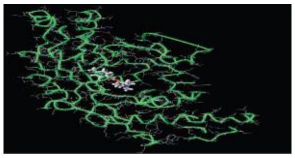

tropine

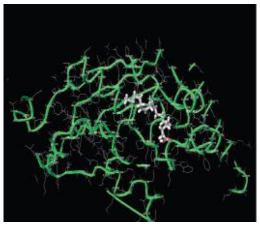

Linoleic acid

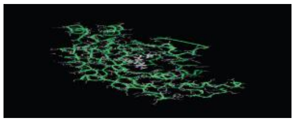

Solacaproine

Figure S1. Molecular docking conformations of selected chemical compounds within the active site of inducible nitric oxide synthase (iNOS).

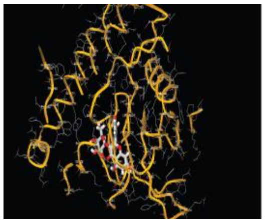

Hyperoside

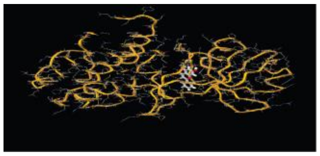

Chrysin

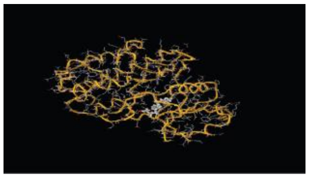

Hexadecanamide

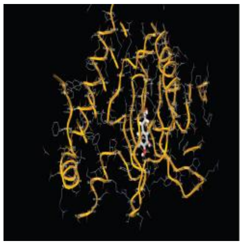

caffeic acid

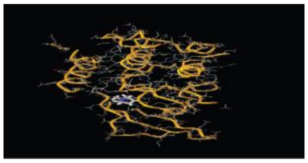

Tropinone

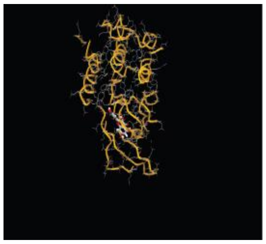

Quercetin

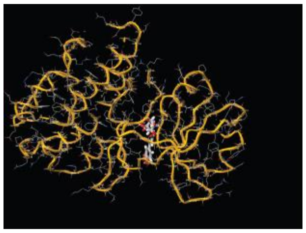

Chlorogenic acid

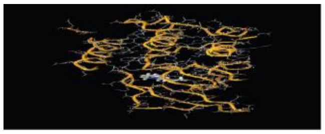

Methylisopelletierine

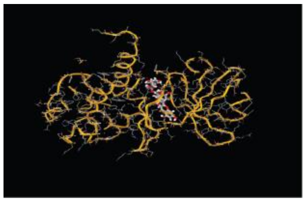

Rutin

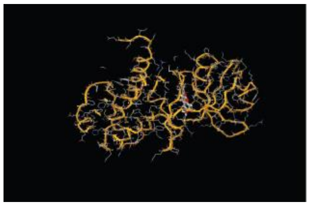

Scopoletin

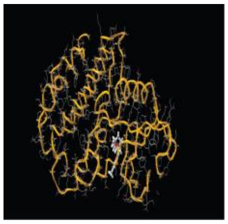

tropine

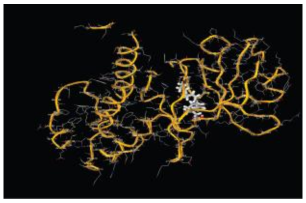

Linoleic acid

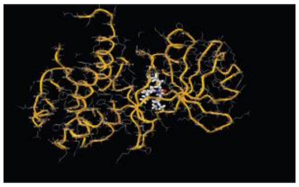

Solacaproine

Figure S2. Molecular docking conformations of selected chemical compounds within the active site of ERK.
